# Human scent as a first-line defense against disease

**DOI:** 10.1038/s41598-023-43145-3

**Published:** 2023-10-04

**Authors:** Amy R. Gordon, Johan N. Lundström, Bruce A. Kimball, Bianka Karshikoff, Kimmo Sorjonen, John Axelsson, Mats Lekander, Mats J. Olsson

**Affiliations:** 1 Division of Psychology, Department of Clinical Neuroscience, Karolinska Institutet, Nobels Väg 9, 171 77 Stockholm, Sweden; 2 Monell Chemical Senses Center, Philadelphia, PA 19104 USA; 3 Lukt och smakmottagningen, Karolinska University Hospital, 141 86 Stockholm, Sweden; 4 Stockholm University Brain Imaging Centre, Stockholm University, 106 54, Stockholm, Sweden; 5 Department of Social Studies, University of Stavanger, 4021 Stavanger, Norway; 6 Stress Research Institute, Department of Psychology, Stockholm University, 106 54 Stockholm, Sweden

**Keywords:** Human behaviour, Immunology, Psychology

## Abstract

Individuals may have a different body odor, when they are sick compared to healthy. In the non-human animal literature, olfactory cues have been shown to predict avoidance of sick individuals. We tested whether the mere experimental activation of the innate immune system in healthy human individuals can make an individuals’ body odor be perceived as more aversive (intense, unpleasant, and disgusting). Following an endotoxin injection (lipopolysaccharide; 0.6 ng/kg) that creates a transient systemic inflammation, individuals smelled more unpleasant compared to a placebo group (saline injection). Behavioral and chemical analyses of the body odor samples suggest that the volatile components of samples from “sick” individuals changed qualitatively rather than quantitatively. Our findings support the hypothesis that odor cues of inflammation in axillary sweat are detectable just a few hours after experimental activation of the innate immune system. As such, they may trigger behavioral avoidance, hence constituting a first line of defense against pathogens of infected conspecifics.

## Introduction

Across phyla, behavior is arguably the first line of defense against infection, preventing or reducing parasite encounter by avoiding contaminated individuals, and is likely the most cost-effective defense^1^. A key component in such a behavioral defense, often referred to as the behavioral immune system, is reactive avoidance of infested individuals^2^. Although sickness cues can be of different sorts, a number of studies now indicate that unhealthy animals can give off an olfactory cue leading to avoidance by conspecifics^3–6^. One influential experimental line of research has made rats sick by injecting them with an endotoxin, lipopolysaccharide (LPS). LPS activates the innate immune system resulting in an inflammatory response. These individuals, and their urine, tend to be avoided by other rats^3,7,8^. Two studies^9,10^ suggested that also humans participants would be able to detect by way of body odor a systemic immune activation following LPS exposure. In parallel with these observations, several studies on visual sickness cues also indicate that subtle visual signs of disease are evident soon after activation of the immune system^11–14^. More recently, we have demonstrated that humans are able to extract multisensory cues of sickness from combined visual and odor cues which interact to elicit superadditive activations of sickness in the brain^15^ and that faces accompanied by sick body odors are less liked^16^.

In the present study, we attempted a replication of our previous work on early olfactory sickness cues detection in humans^9^. In Olsson et al. LPS-exposed participants demonstrated clear evidence of a sickness response (head ache, increase of pro-inflammatory cytokine levels as well as tympanic temperature). Correspondingly, the body odor samples collected from the LPS condition were rated more unpleasant, intense, and unhealthy than healthy samples by other participants. Two possible explanations were considered. Either LPS-exposed donors simply emitted more volatile components or there was a change in the profile of volatiles, a more reliable cue in a natural setting. Two observations argued for the latter. There was a significant effect of LPS-exposure on perceived body odor quality (i.e., unpleasantness) that could not be attributed to a shift in body odor intensity. Moreover, gas chromatography-mass spectrometry (GC–MS) analyses of the body odor samples indicated that the volatile substances in the LPS samples were not more abundant than in the saline samples.

The main aim of the current study was to test whether a lower-grade systemic inflammation (using an experimental LPS dosage of endotoxin that is 25% weaker than previously administered) could also drive a decrease in body odor pleasantness independent of any general shift in body odor intensity. To this end, we administered either low-dose LPS or Placebo (saline) injections to individuals from whom body odors were subsequently sampled. Another group of participants, unaware of the nature of the stimuli, rated the body odor samples for perceived intensity, pleasantness, and disgust. We hypothesized that sick body odors would be perceived as (1) more unpleasant and (2) more intense. We also hypothesized that the shift in unpleasantness cannot be attributed to a shift in intensity. We also investigated whether sick body odors are perceived as more disgusting. In addition, we aimed to assess potential differences in chemical compositions between the two treatment conditions (LPS and Placebo) by GC–MS analyses.

## Method

### Body odor donors and sampling

Fifty healthy volunteers were recruited from the university campus for the donation of body odor in one of two conditions (23 men and 27 women, mean age 28.80 years; SD = 7.20). For inclusion, donors had to be 18–50 years old, right-handed, medication free (except for non-barrier contraceptives for female participants), non-smokers, and without a history of drug abuse, chronic pain, or psychiatric disorders. The donors took part in one session, always at approximately 10 am, during which they received either LPS or saline injections. Twenty-nine (16 women) donors were injected intravenously with 0.6 ng/kg LPS (E. Coli, Lot nr G3E0609, United States Pharmacopeia Rockville, MD), and 21 (11 women) donors were injected with saline in; all in a double-blind procedure and a balanced order. For further details, see Karshikoff et al., in which the sampling was originally done^17^.

Body odors were sampled using tight t-shirts (American Apparel, London, UK; type BB301, 50% cotton, 50% polyester), which were put on directly after the injection and returned to the experimenter at the end of the session and put in freezer (− 20 °C). Donors followed dietary restrictions 12 h before participation and were instructed to sleep for 8 h between 23.00 ± 30 min to 07.00 ± 30 min the night before participation and to avoid spicy food and alcohol as well as odorized products (such as lotions and perfumes) the day prior to and during the session. They received no instructions about shaving their armpits. Donors washed their armpits with a scent free wet wipe before putting on the t-shirt. Urine samples were also collected and studied separately, see Gordon et al.^10^. The sampling took place in a hospital environment and lasted for a total of approximately five hours. A physician supervised the participants during the full sampling phase of the study to ensure immediate medical attention in the event of an acute reaction to the endotoxin.

### Measures of the sickness response

During the sampling period, tympanic temperature (ThermoScan pro 1, Thermoscan, San Diego, CA) was measured before injection and once every hour post-injection. Five hours after injection with LPS, body temperature had increased by about 1 °C. Plasma samples were provided at 0 h (baseline), 1.5 h, 3.5 h and 5 h after injection and were frozen at − 70 °C. Samples were later thawed for analysis with MILLIPLEX MAP high sensitivity human cytokine kit (Millipore, Missouri, USA) with Luminex xMAP methodology. Levels of four cytokines associated with LPS-induced inflammation were measured to confirm an inflammatory response to LPS^18^. These were three proinflammatory cytokines, TNF-alpha, IL-6 and IL-8, and the anti-inflammatory cytokine IL-10. For details, see Karshikoff et al.^17^.

### Chemical analysis

Chemical assays of body odor were conducted in order to assess the relative abundance of potentially odorous (i.e. volatile) compounds with headspace gas chromatography-mass spectrometry (GC–MS). From each donor, two t-shirt pieces (each, a one-sixth sliver of an 11 × 14 cm oval cut from the left and right armpit area) were placed in 20-mL sample vials and subjected to headspace analysis using a HT3 dynamic headspace analyzer (Teledyne Tekmar, Mason, OH, USA) outfitted with Supelco Trap K Vocarb 3000 trap (Sigma-Aldrich Co., St. Louis, MO, USA). The sample vial was maintained at 40 °C, swept with helium for 45 min (flow rate of 75 mL/min), and the volatiles collected on the thermal desorption trap. Trap contents were desorbed at 265 °C directly into a Thermo Scientific ISQ single-quadrupole gas chromatograph-mass spectrometer (Thermo Scientific, Waltham, MA, USA) equipped with a 30 m × 0.25 mm id Stabiliwax-DA fused-silica capillary column (Restek, Bellefonte, PA, USA). The GC oven program had an initial temperature of 40 °C (held for 3.0 min) followed by a ramp of 7.0 °C/min to a final temperature of 230 °C (held for 6.0 min). The mass spectrometer was used in scan mode from 33 to 400 m/z.

Baseline correction, noise elimination, and peak alignment of the chromatographic data were achieved using Metalign^19^. Resulting multivariate data (consisting of all mass spectrometric responses exceeding a defined threshold at each scan event) were further processed with the MSClust tool for mass spectra extraction and generation of individually selected ion chromatogram peak responses^20^. The resultant dataset consisted of a single response for all peaks identified in the chromatograms and was suitable for statistical analyses. To this end, a total of 54 peak responses were tentatively identified by their mass spectra matches to National Institute of Standards (NIST) Standard Reference Database. Of these, we determined that 18 were either related to the chromatographic system or could be identified in chromatograms obtained from unused t-shirts. The remaining 36 peaks relating to the shirts of collected body odorants were retained.

Individual peak responses were normalized by dividing each peak response in the sample by the total of peak responses determined for that sample. This was necessary to account for the variable and unknown mass of sweat collected on the fabric. Average normalized peak responses were then determined for each subject from the analysis of the two fabric pieces and these average responses were subjected to analysis of variance (ANOVA).

### Behavioral assessment of body odor

Body odor perception was assessed in an experiment at Karolinska Institutet, Stockholm, Sweden. Sixty-nine healthy participants (mean age = 25.5; 38 women) were recruited to take part in the body odor assessment experiment. Inclusion criteria were non-smoking, self-reported good health, and self-reported functional sense of smell. In the body odor assessment experiment, participants were tested separately with 52 T-shirt samples presented in 200 mL glass jars (29 LPS + 21 placebo body odor samples + 2 samples from unworn T shirts, Control). Each unique sample consisted of 11 × 14 cm oval cut from the left and right armpit T-shirt area minus the one-sixth sliver that was subject to GC–MS analysis (see above). These 52 odor stimuli were each presented one time in a uniquely randomized order with an inter-stimulus interval of approximately 30 s. The choice to present stimulus only one time and to have a large interstimulus interval was to prevent olfactory fatigue.

After smelling each sample, participants rated its perceived intensity (on a scale between 0 and 7), pleasantness (between − 7 and 7 with 0 representing neutral), and disgust (between 0 and 4). The extremes of these scales were referred to as maximal experiences.

### Ethical permissions and data handling

All methods were performed in accordance with the relevant guidelines and regulations. The study was approved by the Regional Ethical Review Board (2009/892-31/2) in Stockholm. Written informed consent was obtained beforehand from all body odor donors as well as from all participants in the perceptual ratings experiment.

### Statistical analyses

Tests for any effects of treatment on immune response markers were assessed using ANOVA in IBM SPSS Statistics 26. Effects of treatment on ratings of intensity, pleasantness and disgust of body odor were assessed with multi-level analyses in R v.4.1.3^21^. Cross-classified models were used, where intercepts were allowed to vary both between donors and between raters, i.e., they were defined as random. Bayesian estimations of effects of treatment on pleasantness using priors from Olsson et al.^9^ were calculated in R. We define statistical significance at *p* < 0.05. For replications from Olsson et al.^9^ one-sided tests of hypothesized differences were used for changes in body odor percepts.

Average normalized peak responses were subjected to one-factor ANOVA with treatment (LPS or placebo) as fixed effect. The false discovery rate controlling procedure of Benjamini and Hochberg^24^ was used to account for the conduct of multiple tests.

## Results

### Treatment-dependent inflammation responses

Blood plasma cytokine levels from before injection, 1.5, 3.5, and 5 h post injection from LPS- and placebo donors were calculated. Effects of LPS-exposure on cytokine levels were analyzed as the area under the curve (AUC). The group functions for each treatment (AUC as a function of time) for all cytokines peaked within the sampling period, typically between 1.5 and 3 h. The treatment effects were significant as judged by ANOVAs: IL10, *F*(1, 49) = 9.72, *p* = 0.003; TNFα, *F*(1, 49) = 21.18, *p* < 0.001; IL6, *F*(1, 49) = 19.45, *p* < 0.001; and IL8, *F*(1, 49) = 14.79, *p* < 0.001 Thus, the concentration of cytokines following LPS treatment was considerably higher than following placebo treatment. (See Fig. 1 in Gordon et al.^10^ for cytokine responses as a function of time since injection.) Moreover, tympanic temperature also increased more in the LPS group (mean 0.32 ± 3.2 °C) compared to the placebo group (mean 0.02 ± 1.0 °C, *p* = 0.03).

**Figure 1:**
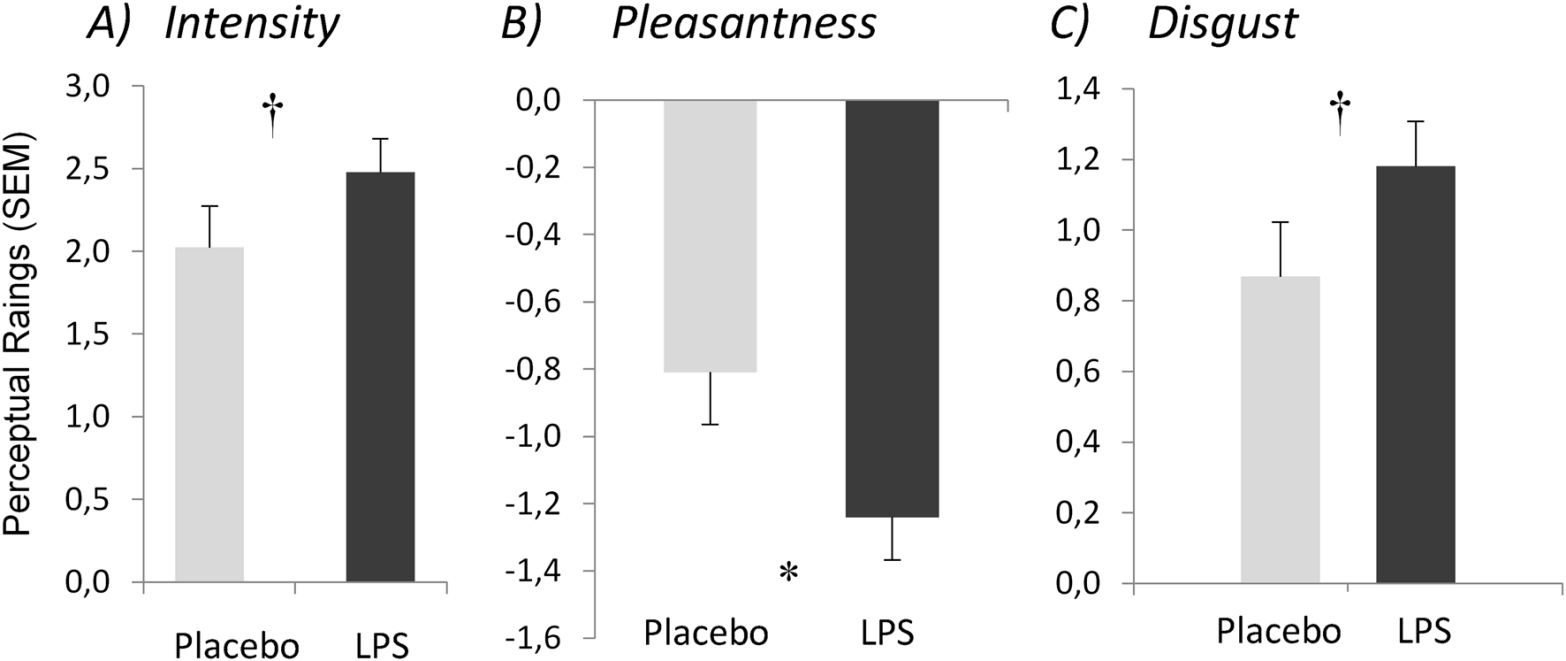
Perceptual ratings of body odor samples. Mean ratings of the perceived (**A**) intensity, (**B**) pleasantness, and (**C**) disgust of T-shirts worn by individuals exposed to lipopolysaccharide (LPS) and a Placebo (saline injection). Error bars show standard errors. An asterisk (*) indicate a significant difference between conditions (p < 0.05) whereas † reflects a p-value between (0.5 and 0.10).

### Body odor perception

LPS body odor smelled more unpleasant than Placebo-treated, Cohen’s *d* = 0.277, *t*(47.929) = 1.716, *p* = 0.047. Although the effect of the LPS treatment was nominally in the hypothesized direction for intensity, *d* = 0.262, *t*(47.909) = 1.459, *p* = 0.075, and for disgust, *d* = 0.243, *t*(47.891) = 2.025, *P* = 0.068, neither was significant (Fig. 1). As in in the previous study^9^ we again tested for a qualitative, treatment-based shift in body odor that was not dependent on the intensity increase. An analysis of the treatment effects on pleasantness ratings, while adjusting for effects of treatment-based intensity increases, yielded a significant decrease, *d* = 0.101, *t*(47.929) = 2.066, *p* < 0.011.

**Figure 2:**
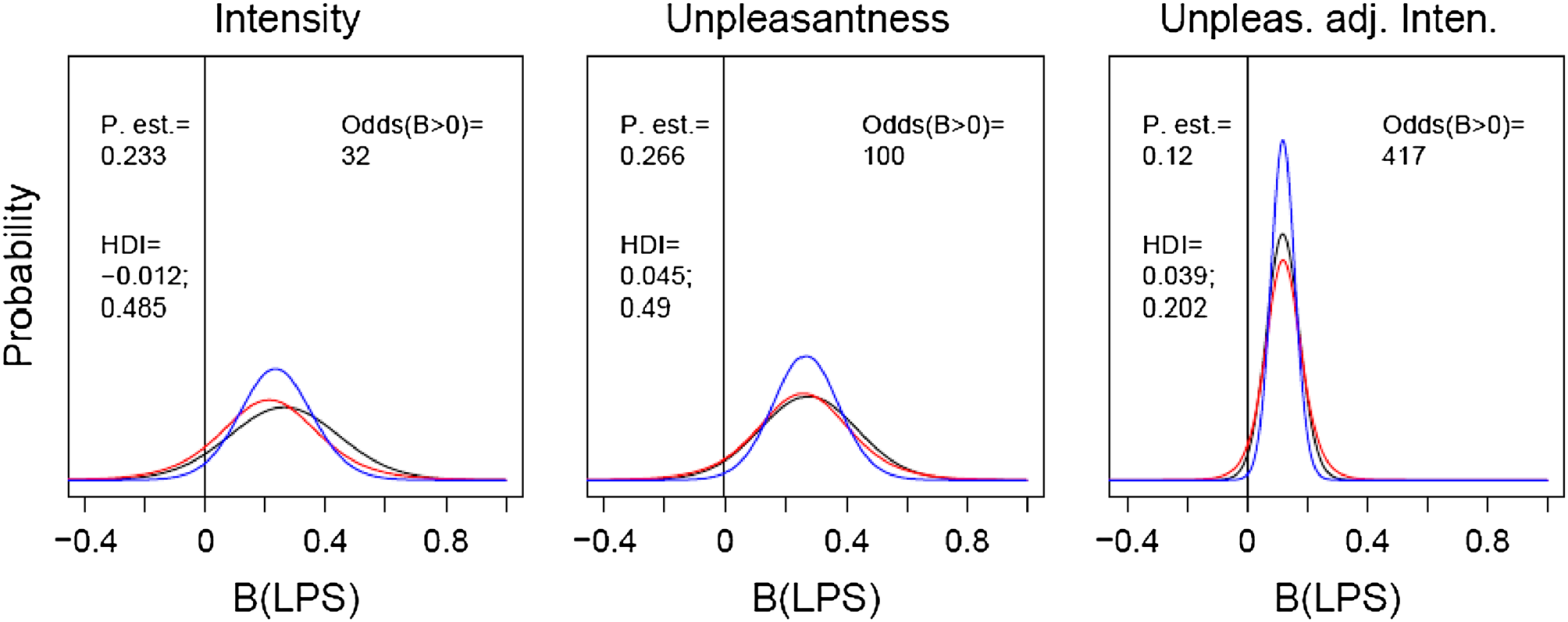
Posterior probability analyses of treatment effects. Likelihood estimates for various effects of LPS treatment in Olsson et al.^9^ (red lines) are used as prior probabilities and multiplied with likelihood estimates in the present study (black lines), which results in a distribution of estimated posterior probabilities (blue lines). Point estimates (P.est; with the highest probability) with Highest Density Interval (HDI; corresponding to confidence interval) for the distribution of posterior probabilities, as well as the estimated odds for an effect-size differing from zero, are included in the plots. Note that the pleasantness scale has been reversed to harmonize the x-axes.

We also analyzed the treatment-dependent shift in body odor ratings utilizing Bayesian statistics and results from Olsson et al.^9^ as priors for intensity, pleasantness, and pleasantness adjusted for intensity. As can be seen in Fig. 2, the evidence for a treatment effect accumulated across these two studies, in the form of the posterior probabilities and their confidence intervals, indicates that LPS-treated body odor does smell more unpleasant and that this effect is not dependent on treatment-based increases in odor intensity (see right-most panel).

### Chemical composition of body odor

GC–MS analyses generated a total of 36 peaks that could potentially be attributed to body odor. Five of these compounds were significantly different between the LPS and placebo samples (Table A). One component, 6-methyl-5-hepten-2-one, endogenous to the body in both human^22^ and non-human animals^23^ was significantly higher after correction for multiple testing^24^ in the LPS compared to the placebo samples (F_1,47_ = 13.12, p = 0.0007).

## Discussion

This study examined whether a lower-grade systemic inflammation could drive a decrease in body odor pleasantness, independent of any general shift in body odor intensity. In line with this aim, individuals who received a 0.6 ng/kg LPS injection had significantly different levels of inflammation-related markers, relative to individuals who received a saline injection—indicating successful experimental activation of the innate immune system and induction of subjective sickness. Further, in line with our expectations and past research, individuals who had received an LPS injection had more unpleasant body odors than individuals in the placebo (saline injection) condition. Moreover, the change in odor pleasantness following an LPS injection is likely driven by chemical composition of body odor, rather than uniform increases or decreases in chemical abundance. Intensity and pleasantness ratings of unpleasant odors are strongly correlated^25^. However, by adjusting for effects of intensity on pleasantness, we have previously demonstrated that LPS treatment decreases pleasantness ratings of body odor, independent of its effects on intensity^9^. This could also be replicated here. Using Bayesian statistics and the results from Olsson et al.^9^ as priors, we calculated the posterior probability by including the current results for the measures that these studies have in common: intensity, pleasantness, and pleasantness adjusted for intensity which gave similar results. Altogether the results support the conclusions of our the previous study^9^, that axillary body odor pleasantness may decrease already within hours of the onset of systemic inflammation. Most importantly, this replication supports the notion that the human sense of smell may be able to detect health-status information and, akin to many other non-human animals, may function as a first line of defense against pathogens.

The central tenet of a behavioral defense against disease is that the detection of sickness in a conspecific can alter patterns of social interaction, either to avoid the sick individual entirely or to trigger behaviors that minimize the risk of contamination^2^. Statistical modeling of social interaction patterns indicates that even minor changes in inter-individual contact patterns can have large disease-containing effects^26^. To allow the behavioral immune system to react, however, an implicit claim is that sensory cues of sickness in others can be detected. As noted, past studies have demonstrated that participants in experimental studies can identify sensory cues of early innate immune system activation via several visual cues, such as skin coloration^12^, gait pattern^14^ and facial features^11,13,16^. Similarly, sensory cues of sickness may interact cross-modally to form superadditive sensory cues of sickness, which further aids the detection of infection^15^. Interestingly, a few studies indicate that not only can disease cues be detected and possibly lead to avoidance, but that they can also activate the immune system in what may be a preparatory response^27,28^. Altogether, these previous findings and the present results support the emerging notion that there is a behavioral defense that promotes avoidance and even interacts with white blood cells to protect humans from contagion. However, whether the established detection and increased averseness of these sensory cues can modify human behavior to *overtly* avoid contact with sick individuals, and whether these potential behavioral modifications are context dependent (and differ based on the identity of the sick individual) remains to be determined immune-defensive behaviors.

The GC–MS peak response of one compound, 6-methyl-5-hepten-2-one, differed significantly in response to endotoxin administration and the subsequent inflammation response. This ketone is a known human sweat component^29^ with dietary and bacterial origins, which has been identified in a study of fecal biomarkers for gastrointestinal disease^22^. While its presence was ubiquitous, its variability allowed for the discrimination of various diseases. The presence of 6-methyl-5-hepten-2-one likely arose from both endogenous (human sweat) and exogenous (fabric substrate) sources. Interestingly, this chemical response does not correlate with the observed body temperature responses of the human subjects receiving the LPS or control treatments. Therefore, the evolution of 6-methyl-5-hepten-2-one cannot merely be attributed to increase temperature and moisture (sweat) effects on the fabric. It is possible that increased 6-methyl-5-hepten-2-one was partly responsible for the behavioral results. However, these data do not provide direct causal links between the chemical and perceptual changes.

A previous publication on detectible, inflammation-induced changes in human urine odor identified LPS-dependent variability in abundance of the volatile compounds pyrrole and acetophenone^10^. Of these we could identify acetophenone here, but no effect of LPS. In the current study on the same donors, our analyses instead identified 6-methyl-5-hepten-2-one. Even though samples for the two studies were obtained from the same participants, what sets the two studies apart is the difference in odor sources—axillary sweat vs. urine. It is well known that presence and abundance of human volatile compounds in human varies among secretions^30^. That we are able to replicate the behavioral findings in two studies, using two LPS doses differing in concentration (0.6 vs. 0.8 ng/kg) supports the existence of some chemical signal that mediates differences in perceptual ratings. However, it is likely that the combined limitations of these types of studies, including natural variation in chemical excretion by treated participants, potential insensitivities towards certain chemical compounds by the sampling media and methods, and potential external contamination of the sampling media by natural contaminants in the air and manufacturing of the t-shirts necessitate a much larger sample in order to identify changes in individual peaks with high statistical certainty.

The olfactory system is more attuned to patterns in chemical composition rather than isolating and processing individual chemicals^31^. Indeed, identified body odor signals are commonly comprised of complex mixtures^32,33^. Mice have been demonstrated to be highly attuned to pattern identification or combinatorial odor codes and are skilled at identifying these patterns even when masked by other odor sources. For example, chemical signals that allow an animal to identify a conspecific based on their urine alone are not obfuscated by any large dietary changes that trigger profound alterations in urinary chemical composition^34^. Moreover, 50-years of studies attempting to identify what chemical compounds allow animals, and potentially humans^35^, to identify or differentiate individuals based on their MHC composition alone have demonstrated that while no individual chemical compounds can explain this ability, the animals are extracting some form of information from sampled body odors^36,37^. Based on these animal data and the inconsistencies between our own human studies, future experimental analyses of larger samples should focus on the recognition of odorant patterns using methods allowing for the identification of changes in complex patterns, such as support-vector or random forest machine learning methods.

The fact that we used a lower dose of LPS, relative to the previous study^9^, is a strength of this study, but also poses challenges. Immune markers demonstrated that LPS clearly activated donors’ immune systems, but verbal assessments of their degree of illness indicated that they felt less physically ill than the donors receiving a larger LPS dose. The low dose may partially explain why the effect size of the pleasantness ratings difference was low-to-medium. While it is interesting to note that even a lower dose of endotoxin results in a detectable chemical signal of sickness, it makes for less than ideal replication conditions. Moreover, the between-groups design means that there is a possibility that the observed perceptual differences are derived from an inherent difference in donor groups.

Our results suggest that a systemic inflammation response makes body odors more unpleasant than those collected from healthy participants and that this effect does not seem to rely on a general shift in the abundance of body odor volatiles. Taken together, these findings constitute a direct replication of Olsson et al.^9^, and a conceptual replication of Gordon et al.^10^, and lend further support to the notion that humans, like their animal counterparts, are able to detect chemical cues of sickness, a requisite component of a behavioral defense against disease. Whether these effects would translate to changes in overt behavior, such as avoidance, remains to be experimentally assessed.

### Supplementary Information

Supplementary Table 1.

## Data Availability

The datasets used and analyzed during the current study are available from the corresponding author on request.
