# Supplementary material for: Human scent as a first-line defense against disease
Source: Sci Rep. 2023 Oct 4;13:16709. doi: 10.1038/s41598-023-43145-3 (PMC10550911; doi:10.1038/s41598-023-43145-3)
Supplement: Supplementary file 1 — Supplementary Table 1. [file 41598_2023_43145_MOESM1_ESM.docx]

Table 1. Tentative identifications of compounds of likely endogenous origin that differed significantly in average normalized peak responses between the LPS and Placebo groups. Asterisk (*) indicates a p-value that sustains correction for multiple testing. See method section for further information.

**______________________________________________________________**

**Compound P-value LPS/Placebo Placebo LPS**

**______________________________________________________________**

6-methyl-5-hepten-2-one .00071* 1.51 0.06924 0.104733

3-octen-2-one .01470 1.21 0.020438 0.024731

1-methoxy-2-propanol .01695 0.71 0.030335 0.021606

Octanal .01695 1.39 0.006605 0.009164

1-butanol .02835 0.82 0.270376 0.223021

______________________________________________________________
